# Value of M2BP in predicting in‐stent restenosis in patients after coronary drug‐eluting stent implantation

**DOI:** 10.1002/clc.23775

**Published:** 2022-01-15

**Authors:** Le Yang, Haijun Zhu, Yuanyuan Sun, Pengcheng Yan, Xiaoning Song, Fayun Xu, Haitao Yuan, Liming Chen

**Affiliations:** ^1^ Department of Cardiology Shandong Provincial Hospital Affiliated to Shandong First Medical University Jinan Shandong China; ^2^ Department of Cardiology, Shandong Provincial Hospital, Cheeloo College of Medicine Shandong University Jinan Shandong China; ^3^ Department of Cardiology ZiBo Central Hospital Zibo Shandong China; ^4^ Department of Geriatric Cardiology Shandong Provincial Hospital Affiliated to Shandong First Medical University Jinan Shandong China

**Keywords:** inflammation, in‐stent restenosis, mac‐2 binding protein, migration, proliferation, vascular smooth muscle cells

## Abstract

**Objective:**

We evaluated the association between plasma levels of mac‐2 binding protein (M2BP) with the risk of in‐stent restenosis (ISR) after percutaneous coronary intervention (PCI).

**Methods:**

Plasma M2BP levels were compared between 258 patients who experienced ISR at 12‐months post‐PCI and 258 patients, matched for age and sex, without angiographic evidence of ISR.

**Results:**

The plasma M2BP level was significantly higher in the ISR than in the non‐ISR group. On multivariate analysis, adjusted for potential clinical, biochemical, and angiography characteristics, M2BP remained as an independent significant predictor of ISR.

**Conclusions:**

M2BP may be an important predictive biomarker of ISR and may be useful in identifying at‐risk patients.

## Summary Points

- In‐stent restenosis (ISR) remains a major problem after percutaneous coronary intervention (PCI) and the underlying mechanisms have not yet been fully elucidated.
- Plasma Mac‐2 binding protein (M2BP) levels were evaluated in 258 patients who experience ISR and 258 patients without angiographic evidence of ISR at approximately 12 months after PCI.
- Notably, plasma M2BP levels were significantly higher patients with (12.91 ± 5.17 μg/ml) than without (10.07 ± 4.86 μg/ml) ISR (*p* = .001).
- Compared with patients with the lowest tertile of M2BP, patients in the mid and highest tertiles of plasma M2BP had a more than 1.3‐fold and 1.8‐fold increased risk of ISR, respectively (both *p* < .001).
- Multivariate logistic regression analysis revealed that, after adjustment for potential clinical factors, elevated plasma M2BP remained an independent predictor of ISR, either as a continuous or as a categorical variable (both *p* < .001).
- M2BP is an important predictive biomarker of ISR and a mediator of growth and migration in vascular smooth muscle cells.
- Plasma M2BP assessment may be useful in identifying patients who are at high risk of ISR.

## 1 INTRODUCTION

Percutaneous coronary intervention (PCI) using drug‐eluting stent (DES) implantation has revolutionized the treatment of patients with coronary artery disease (CAD).^1^, ^2^ However, in‐stent restenosis (ISR) of the target vessel, resulting from the irreversible mechanical damage to the vascular intima during the process of PCI, has hampered its long‐term efficacy.^3^ The pathophysiology of ISR has not yet been well elucidated. It has been postulated that the inflammatory response resulting from injury to the vessel during PCI triggers and organizes the process of ISR through activation of multiple pathways, such as the Notch1, platelet‐derived growth factor type BB (PDGF‐BB)/ROS/NF‐kappa B/mTOR/P70S6K, endothelial cell‐specific SOD‐1/RhoA/JNK, transforming growth factor (TGF)‐beta/bone morphogenic protein, Hedgehog, and cytokine/chemokine‐related inflammatory signaling pathways.^3^, ^4^, ^5^, ^6^, ^7^

Mac‐2 binding protein (M2BP) is a secreted glycoprotein belonging to the macrophage scavenger receptor cysteine‐rich domain superfamily.^8^ M2BP is widely expressed in human tissues, including the lungs, stomach, and colon, and has also been identified in human fluid, including urine, tears, saliva, breast milk, and plasma.^9^ An elevated baseline level of M2BP expression is associated with poor survival among patients with various types of cancer.^9^ Previous studies also revealed a pivotal role of elevated M2BP in inflammatory diseases, including hepatic fibrosis, chronic pancreatitis, bronchial asthma, and venous thrombosis.^10^, ^11^, ^12^ The pro‐inflammatory characteristics of M2BP were further revealed by an in vitro study which revealed the stimulation role of M2BP in increasing interleukin (IL)‐2 production in peripheral blood mononuclear cells and IL‐6 expression by bone marrow stroma cells.^13^ Furthermore, a recent study showed that M2BP was expressed in pro‐inflammatory M1 macrophage in‐vitro and colocalized with human plaque macrophages in vivo.^14^ More recent studies described the clinical relationship between M2BP and coronary artery disease (CAD). Observational research revealed that the plasma M2BP level was independently associated with long‐term mortality among patients with CAD, where CAD was confirmed by coronary computed tomography angiography.^15^ In addition, our newly published study showed that the plasma M2BP level might be a predictor of vulnerable plaque, as well as being an independent predictive factor of poor cardiovascular outcomes among patients with acute coronary syndrome (ACS).^16^ These results infer a possible role of M2BP in atherosclerosis development and plaque instability.

Given the pro‐inflammatory role of M2BP and mounting evidence implying its potential correlation with atherosclerosis, we speculated that M2BP might be potentially involved in the process of ISR. To test this hypothesis, we compared the plasma M2BP level in consecutive patients with angiographically documented ISR to those of patients who did not develop ISR after DES‐based PCI.

## 2 METHODS

### 2.1 Study population

Eligible were the 3099 patients with ACS who underwent baseline coronary angiography (CAG) and subsequent DES‐based PCI of de novo lesions in native coronary arteries, between October 2014 and June 2018, in the Department of Cardiology, Shandong Provincial Hospital, which is affiliated to Shandong First Medical University, Jinan, Shandong, China. The following patients were excluded: lost to follow‐up (*n* = 182); unwilling to undergo follow‐up angiography (*n* = 225); and confirmed death (*n* = 50). This left 2642 patients with follow‐up data at 12 months post‐PCI. Of these, 673 underwent a planned follow‐up CAG or CAG, at 3–12 months after the PCI procedure, due to recurrent symptoms or abnormal noninvasive test results for angina, either treadmill exercise tests or myocardial perfusion scintigraphy. After exclusion of cases of in‐stent thrombosis (*n* = 3), ISR was identified in 308 cases. ISR was defined as recurrence of luminal diameter stenosis >50% within the stent or within adjacent segments, 5‐mm proximal or distal to the stent, observed on follow‐up angiography.^16^ Of the 308 patients who were diagnosed with ISR, those who had concomitant valvular disease (*n* = 8), systematic inflammatory disease (*n* = 16), malignant tumor (*n* = 10), severe liver disease (*n* = 6), or moderate‐severe chronic renal insufficiency (eGRF < 60 ml/min/1.73 m^2^, *n* = 10) were excluded. The remaining 258 patients with ISR formed our study cohort. We also randomly selected another 258 age‐ and sex‐matched patients who had no ISR on follow‐up CAG, within the same study period as the control group.

All patients provided written informed consent. The protocol followed the principles of the Declaration of Helsinki and was approved by our institutional review board.

### 2.2 Coronary angiography and analysis

Coronary angiography was performed according to the standard Judkins technique. Quantitative evaluation of coronary angiography was performed, before the procedure and at the 12‐month follow‐up, by two cardiologists who were blinded to the study protocol and to patient information. Using the outer diameter of the contrast‐filled catheter as a reference, the single “worst” view, among multiple projections, was recorded as the minimal lumen diameter (MLD). Lesion length was quantified, proximal and distal to the point of MLD, on the projection with the least amount of foreshortening. An MLD value of 0 mm was regarded as a total occlusion at baseline.

Stents were implanted via a normal‐to‐normal technique, using normal segment, 5‐mm in length, proximal and distal to the target lesion. The late loss was defined as the difference between the MLD measured immediately after the procedure and the MLD quantified on follow‐up angiography. In patients with multiple coronary lesions, the lesion with the greatest late loss was entered in the analysis.

### 2.3 Biochemical analysis and measurement of plasma M2BP

Peripheral blood samples were collected at the time of admission for coronary angiography, after overnight fasting. Blood samples to quantify the M2BP level were collected in tubes containing potassium EDTA; samples were centrifuged for 15 min at 2000 rpm and stored at −80°C until analysis. The plasma M2BP level was quantified using the Human s90K/Mac‐2BP Platinum ELISA (eBioscience), according to the manufacturer's instructions. All measurements were performed in duplicate and in blinded fashion. Plasma levels of fasting glucose, creatinine, low‐density lipoprotein‐cholesterol (LDL‐C), high‐density lipoprotein cholesterol (HDL‐C), total cholesterol, and triglycerides were quantified using standard laboratory procedures in the Department of Clinical Laboratory in Shandong Provincial Hospital.

### 2.4 Statistical analysis

The normality of the distribution of data was assessed using the Kolmogorov–Smirnov test. Continuous variables were reported as the mean ± standard deviation (*SD*), with between‐group differences evaluated using an unpaired Students' *t*‐test. Categorical variables were reported as counts and percentages, with between‐group differences evaluated using a *χ* ^2^ test. Patients with ISR were categorized into three groups according to the tertile distribution of plasma M2BP level (µg/ml). Multivariable logistic regression models were constructed to detect the relationship between ISR and plasma concentration of M2BP. Factors that were statistically significant (*p* < .1) on univariate analysis and those known to be clinically relevant were included in the final multivariate logistic model to identify independent predictors of ISR, as described in our previous study.^16^ All tests were two‐tailed, with a *p*‐value <.05 considered significant. All analyses were performed using SPSS (version 18.0 for Windows; SPSS).

## 3 RESULTS

The baseline clinical, laboratory and angiographic data for the study cohort are detailed in Table 1. Compared with the control group, the ISR group had a higher proportion of patients with a family history of CAD, as well as a higher proportion of cigarette smoking, the incidence of dyslipidemia, hypertension, and diabetes mellitus. Moreover, patients with ISR tended to have worse renal function, higher fasting glucose, and higher total and LDL cholesterol but lower HDL cholesterol levels than those without ISR. At the 12‐month follow‐up, there were no significant differences between the ISR and non‐ISR groups with regard to age, sex, ejection fraction, and medical treatments.

**Table 1:** Baseline clinical, biochemical, and angiographic characteristics of study subjects

	ISR (−) (*n* = 258)	ISR (+) (*n* = 258)	*p* value
Age (years)	59.1 ± 10.4	61.8 ± 10.1	.569
Men/women	178/80	183/75	.631
Systolic blood pressure (mm Hg)	137.6 ± 18.2	139.8 ± 20.7	.329
Diastolic blood pressure (mm Hg)	85.1 ± 10.1	84.7 ± 12.8	.239
Family history of CAD (*n*, %)	33 (12.8)	68 (26.4)	<.001
Cardiovascular risk factors			
Hypertension (*n*, %)	22 (8.5)	181 (70.2)	<.001
Diabetes mellitus (*n*, %)	40 (15.5)	65 (25.2)	.006
Hyperlipidemia (*n*, %)	19 (7.4)	151 (58.5)	<.005
Current smoker (*n*, %)	71 (27.5)	109 (42.2)	<.001
Biochemistry			
Total cholesterol (mmol/L)	4.85 ± 1.09	4.96 ± 1.13	<.005
LDL‐cholesterol (mmol/L)	2.77 ± 0.83	2.91 ± 1.54	<.001
HDL‐cholesterol (mmol/L)	1.25 ± 0.31	1.13 ± 0.28	<.001
Triglyceride (mmol/L)	1.93 ± 1.51	1.96 ± 1.75	.326
White blood cell (×10^9^/L)	7.82 ± 1.51	7.22 ± 2.08	.369
Fibrinogen (g/L)	3.11 ± 1.17	3.22 ± 0.91	.129
Fasting glucose (mmol/L)	5.92 ± 1.42	6.24 ± 1.96	<.001
GFR, ml/min/1.73m^2^	94.12 ± 17.58	82.33 ± 16.89	<.001
M2BP (ug/ml)	10.07 ± 4.86	12.91 ± 5.17	.001
Ejection fraction, %	62.58 ± 5.57	61.14 ± 7.48	.458
Cardiac medication after PCI			
Dual antiplatelet therapy (*n*, %)	253 (98.1)	257 (99.6)	.239
Beta‐blockers (*n*, %)	190 (73.6)	220 (85.3)	.369
ACEI/ARB (*n*, %)	130 (50.4)	152 (58.9)	.296
Calcium antagonists	105 (40.7)	102 (39.5)	.445
Statins (*n*, %)	245 (95)	255(98.8)	.368
Coronary angiography			
Gensini score	62.4 ± 31.16	62.2 ± 36.9	.129
Lesion characteristics			
CTO	21 (8.1)	55 (21.3)	<.005
Bifurcation lesion	53 (20.5)	83 (32.2)	<.001
LM lesion	16 (6.2)	16 (6.2)	.364
Stent characteristics			
Stent number	1.9 ± 1.1	2.1 ± 1.3	.042
Stent diameter (mm)	2.97 ± 0.33	2.86 ± 0.39	<.001
Total stent length (mm)	40.42 ± 16.89	43.89 ± 17.56	.023
Stent type			
Sirolimus	169 (65.5)	176 (68.2)	.513
Zotarolimus	53 (20.5)	48 (18.6)	.579
Everolimus	20 (7.8)	16 (6.2)	.489
Tacrolimus	16 (6.2)	18 (7.0)	.723

*Note*: Data shown are *n* (%) or mean ± *SD*.

Abbreviations: ACEI, angiotensin‐converting enzyme inhibitors; ARB, angiotensin receptor blockers; CAD, coronary artery disease; CTO, chronic total occlusion; GFR, glomerular filtration rate; HDL, high‐density lipoprotein; ISR, in‐stent restenosis; LDL, low‐density lipoprotein; LM, left main coronary artery; M2BP, mac‐2 binding protein; PCI, percutaneous coronary intervention; *SD*, standard deviation.

Although the degree of coronary stenosis before PCI, the occurrence of left main coronary artery (LM) stenosis, and type of DES implanted were similar between the ISR and non‐ISR group, complicated coronary lesions, such as coronary chronic total occlusions (CTO) (*p* < .005) and bifurcation lesions (*p* < .001), were more frequent in the ISR than the non‐ISR group. Moreover, patients with ISR tended to have a greater number of stents implanted and a greater total length of stenting, but smaller stent diameter, than the non‐ISR group (Table 1).

**Figure 1:**
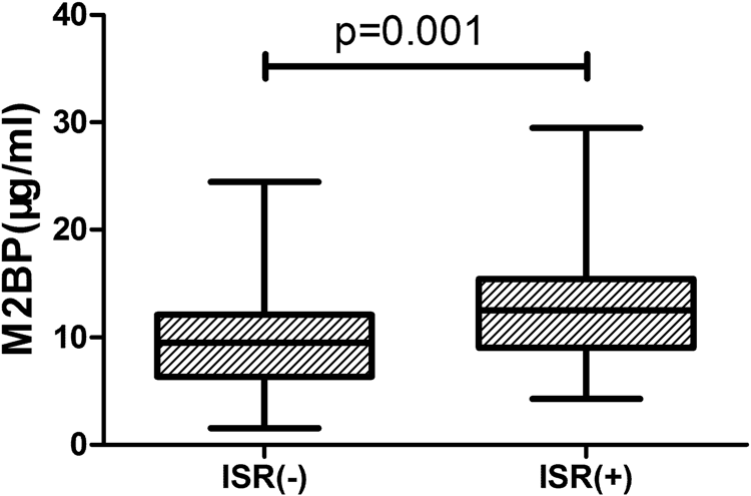
Comparison of plasma levels of mac‐2 binding protein (M2BP) between patients with and without in‐stent restenosis (ISR)

**Figure 2:**
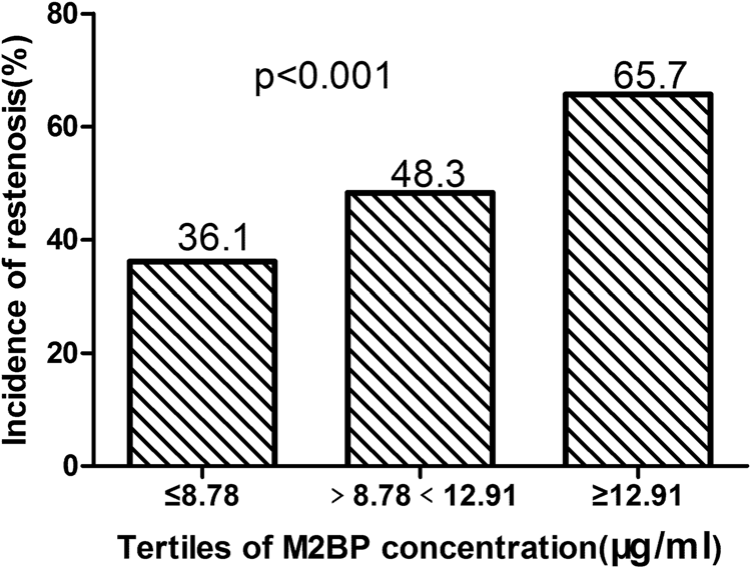
Incidence of in‐stent restenosis (ISR) according to the tertiles of plasma concentration of mac‐2 binding protein (M2BP)

**Figure 3:**
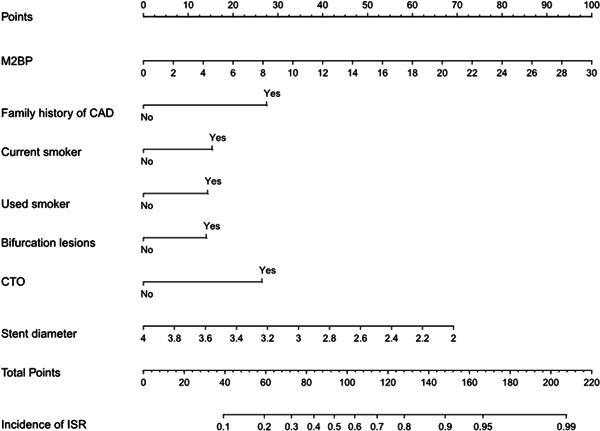
Nomogram model of ISR‐free probability. CAD, coronary artery disease; CTO, chronic total occlusion; ISR, in‐stent restenosis; M2BP, mac‐2 binding protein

Plasma levels of M2BP were considerably higher in the ISR (12.91 ± 5.17 μg/ml) than non‐ISR (10.07 ± 4.86 μg/ml) group (*p* = .001; Table 1; Figure 1). There was a stepwise increase in the incidence of ISR from the lowest tertile (<8.78 μg/ml) to the highest tertile (<12.91 μg/ml) plasma M2BP level (*p* < .001; Figure 2). On multivariate logistic regression analysis, after adjusting for potential clinical, biochemical, and angiography characteristics, an elevated level of M2BP remained as an independent significant predictor of ISR, either as a continuous variable (odds ratio 1.221; 95% confidence interval, 1.143–1.305, *p* < .001) or as a categorical variable (odds ratio 3.722; 95% confidence interval, 2.314–5.987; *p* < .001; Table 2). Prognostic nomograms were also built, based on multivariate Cox regression analysis, using all significant independent indicators of ISR. Nomograms provided an individualized 12‐month probability risk of ISR, assuming the patient did not die of other causes within this 12‐month period (Figure 3).

**Table 2:** Multivariate logistic regression analysis for independent predictors of ISR in patients studied

	Adjusted OR (95% confidence interval)	*p* value
M2BP as a continuous variable (Model I)		
M2BP	1.221 (1.143–1.305)	<.001
Family history of CAD	3.275 (1.923–5.580)	<.001
Current smoking	2.097 (1.360–3.236)	.001
Bifurcation lesions	1.627 (1.004–2.635)	.048
CTO	2.797 (1.542–5.075)	.001
Stent diameter	0.25 (0.136–0.462)	<.001
M2BP as a categorical variable (Model II)		
M2BP > 10.9 μg/ml (median value)	3.722 (2.314–5.987)	<.001
Family history of CAD	2.875 (1.698–4.869)	<.001
Current smoking	2.311 (1.501–3.556)	<.001
Bifurcation lesions	1.616 (1.001–2.611)	.050
CTO	2.619 (1.445–4.747)	.002
Stent diameter	0.246 (0.134–0.450)	<.001

*Note*: In Model I and Model II, adjusted covariates also included age, gender, diabetes mellitus, hypertension, hyperlipidemia, GFR, ejection fraction, stent number, and total stent length.

Abbreviations: CAD, coronary artery disease; CTO, chronic total occlusion; GFR, glomerular filtration rate; ISR, in‐stent restenosis; M2BP, mac‐2 binding protein; OR, odds ratio.

## 4 DISCUSSION

Importantly, our study shows that an elevated plasma level of M2BP at baseline is associated with an increased risk for restenosis at 12 months after coronary stent placement.

Since the introduction of percutaneous transluminal coronary angioplasty (PTCA) in 1977, restenosis, which has an occurrence rate of 32%–40% at 6 months after PTCA, has hampered the long‐term efficacy of PTCA.^2^, ^17^ Use of drug‐eluting stents (DES), which are coated with antiproliferative drugs, such as sirolimus or paclitaxel, has dramatically reduced the occurrence rate of ISR.^18^ However, despite the use of dual antiplatelet and intensive statins therapy, the incidence of ISR after PCI remains high.^19^ Although many drugs and devices have been evaluated to lower the rate of restenosis in humans, no clinical trials have demonstrated a definite benefit of these in preventing or even reducing ISR to date. Therefore, studying the mechanisms of restenosis is needed to explore possible new preventative treatments.^20^ According to recent studies, abnormal proliferation and migration of vascular smooth muscle cells (VSMCs) and chronic inflammation, regulated by various inflammatory factors, are the major pathological processes underlying the development of ISR after stent placement.^3^, ^4^, ^21^

M2BP, which circulates abundantly in plasma, exerts pathogenic effects on arteries when its level is significantly increased, as shown in our study and previous research.^11^ As a member of the macrophage scavenger receptor cysteine‐rich domain superfamily, M2BP was originally identified as a tumor‐associated glycoprotein associated with tumor progression and metastasis.^9^ An elevated expression of M2BP was also identified in inflammation‐related diseases, such as venous thrombosis, asthma, and chronic pancreatitis.^10^, ^11^, ^12^ Recently, the pro‐inflammatory effect of M2BP was further described by in vitro studies, showing its capacity to induce production of IL‐2 via peripheral mononuclear cells and IL‐6 by bone marrow stromal cells.^13^ Shaked et al. further demonstrated that M2BP is highly expressed and secreted by pro‐inflammatory M1 macrophage in vitro and colocalized with human plaque macrophages in vivo.^14^ Evidence of the clinical association between M2BP and CAD has recently emerged. Results from a retrospective study revealed that the concentration of circulating M2BP was independently associated with the long‐term outcome of patients with CAD.^14^ Our own previous clinical observations showed that the plasma levels of M2BP reflected the stability of coronary atherosclerotic plaque, characterized by inflammation in the vessel wall, and, thus, may predict adverse outcomes among patients with ACS.^22^ In a more recent study, we further demonstrated that M2BP was highly expressed in unstable human carotid artery plaques and vulnerable regions within plaques, as well as being significantly correlated to clinical ischemic manifestations.^23^ As atherosclerosis and vascular remodeling share common mechanisms of chronic inflammation and endothelial injury, we postulate that assessment of the plasma level of M2BP may be effective in reflecting the severity of vascular inflammation after injury in a similar way. This is supported by our current findings that an elevated baseline level of M2BP predicted an increased risk of ISR at the 12‐month follow‐up.

The exact mechanisms by which an elevation in M2BP increases the risk of ISR remain largely unknown although several possibilities have been considered, as follows. First, in‐vitro studies have shown that the M2BP protein promotes the migration and proliferation of VSMCs in a dose‐dependent fashion, which is pivotal in the process of neointimal formation and ISR after mechanical injury to the vascular intima. Second, previous studies have uncovered that M2BP can stimulate the expression and secretion of many pro‐inflammatory factors, including IL‐6 and TNF‐alpha) by monocyte‐derived macrophages. This is important as vascular inflammation after stent implantation and endothelial denudation are characterized by infiltration of monocytes and macrophages, as well as production of inflammatory mediators and growth factors. This, in turn, stimulates the aggregation of VSMCs and the deposition of extracellular matrix, resulting in intimal hyperplasia and ISR.^24^ These M2BP‐induced biological effects are crucial to the pathophysiology of vascular remodeling in ISR. Collectively, these results provide potential evidence to support the results of our current observation that M2BP may exert pathogenic effects of ISR by promoting vascular inflammation, migration, and proliferation of VSMCs. However, further research and increased clinical evidence are needed to elucidate the exact mechanisms of ISR and the role of M2BP.

The limitations of our study need to be acknowledged in the interpretation of results. First, our study is limited by its single‐center and cross‐sectional design, as well as by the relatively small sample size. Second, owing to the retrospective design, the causal relationship between M2BP and ISR could not be defined; further in vivo studies, using animal models, are warranted to fully elucidate the mechanisms of restenosis after PCI. Third, the molecular mechanism by which M2BP exerts biological effects on VSMCs still needs further exploration.

## 5 CONCLUSION

An elevated plasma level of M2BP at baseline appears to be associated with an increased risk of ISR at 12 months after PCI. Measurement of M2BP may be helpful to identify patients undergoing PCI who are at high risk of restenosis.

## CONFLICT OF INTERESTS

The authors declare that there are no conflicts of interest. We certify that none of the authors have any financial and/or nonfinancial relationships with an organization or entity with a financial interest in or financial conflict with the subject matter or materials discussed in the manuscript.

## AUTHOR CONTRIBUTIONS

Liming Chen and Haitao Yuan designed the study and wrote the protocol. Le Yang and Haijun Zhu performed most of the research and drafted the manuscript. Yuanyuan Sun and Fayun Xu managed the literature searches and screening. Pengcheng Yan and Xiaoning Song contributed to analyses and statistical interpretation. All authors contributed and have approved the final manuscript.

## PATIENT CONSENT STATEMENT

For investigations involving human subjects, informed consent has been obtained from the participants involved.

## Data Availability

All data included in this study are available upon request by contact with the corresponding author.
